# Exploring training transfer in neurosurgical specialty nurse education: a qualitative descriptive study

**DOI:** 10.3389/fneur.2026.1915268

**Published:** 2026-09-11

**Authors:** Shuang Liang, Na Zhang, Yimin Jiang, Jingyu Li, Zhiying Shang, Cuiling Ji, Lu Chen

**Affiliations:** 1 School of Nursing, Nanjing University of Chinese Medicine, Nanjing, Jiangsu, China; 2 Department of Nursing, Research Institute, Nanjing University Medical School Affiliated Nanjing Drum Tower Hospital, Nanjing, Jiangsu, China; 3 School of Nursing, Nanjing Medical University, Nanjing, Jiangsu, China; 4 Department of Neurosurgery, Nanjing University Medical School Affiliated Nanjing Drum Tower Hospital, Nanjing, Jiangsu, China; 5 Department of Neurosurgery, Nanjing Drum Tower Hospital Clinical College of Nanjing University of Chinese Medicine, Nanjing, Jiangsu, China

**Keywords:** continuing nursing education, neurosurgical nursing, qualitative research, specialty nurse education, training transfer

## Abstract

**Objective:**

To explore the characteristics and influencing factors of training transfer among neurosurgical specialty nurses, and to provide empirical evidence for nursing educators, clinical managers, and training designers to develop optimization strategies.

**Methods:**

Purposive sampling recruited 32 participants (15 specialty nurses, 11 instructors, and 6 training managers) from 16 Chinese institutions. Semi-structured interviews were conducted, and data were analyzed using framework analysis. This study followed the Consolidated Criteria for Reporting Qualitative Research.

**Results:**

Two overarching themes and eight subthemes were identified. Theme 1 described the practical manifestations of training transfer among neurosurgical specialty nurses, including effective transfer of basic perioperative care competencies, adaptive application of advanced competencies, and extension of training outcomes from individual care to specialty development. Theme 2 identified facilitators and barriers influencing training transfer, including individual motivation and professional identity, contextualized teaching, organizational empowerment, resource mismatch, and sustainability challenges after training.

**Conclusion:**

Training transfer among neurosurgical specialty nurses represents an adaptive reconstruction process shaped by individual readiness, educational design, organizational support, and specialty-specific clinical contexts. Effective transfer requires not only high-quality training but also appropriate role positioning, resource alignment, and sustained post-training support.

## 1 Introduction

Neurosurgical patients often present with complex and rapidly changing conditions and frequently experience neurological impairment, consciousness disturbance, seizures, and multisystem complications during the perioperative period. These conditions place high demands on nurses’ specialty assessment, clinical decision-making, and continuous care competencies. With the development of neuroimaging, neuromonitoring, precision treatment, and enhanced recovery concepts, neurosurgical nursing has gradually shifted from traditional postoperative observation toward a specialized nursing model centered on risk warning, evidence-based decision-making, multidisciplinary collaboration, and full-cycle management (1, 2). The World Health Organization emphasizes the importance of strengthening nursing workforce development and enhancing nurses’ capacity to respond to complex health challenges in its State of the World’s Nursing report (3). Against this background, neurosurgical specialty nurse training has become an important strategy for improving specialty nursing quality and patient safety.

However, training effectiveness does not necessarily equate to changes in clinical practice. Whether specialty nurses can effectively apply and sustain the knowledge, skills, and professional concepts acquired during training in real work settings is referred to as “training transfer,” which serves as a key link between continuing education investment and clinical practice transformation (4). Baldwin and Ford proposed that training transfer is jointly influenced by multiple factors, including learner characteristics, training design, and the work environment (4). In recent years, nursing studies have gradually shown that training transfer depends not only on knowledge acquisition but also on organizational support, practice opportunities, role authorization, and team collaboration (5–8). Particularly in highly complex and context-dependent specialty fields, translating training content into stable nursing behaviors remains a considerable challenge.

Neurosurgical nursing is highly dynamic and complex. Patients’ conditions may deteriorate rapidly within a short period, and nursing work involves neurological monitoring, emergency identification, rehabilitation guidance, psychological support, and multidisciplinary collaboration. Specialized roles such as neurosurgery nurse coordinators further emphasize the importance of interdisciplinary coordination and continuous care management in improving the quality of neurosurgical care (9–11). Compared with general nursing training, training transfer among neurosurgical specialty nurses is more likely to be influenced by contextual factors such as case resources, technical platforms, organizational environments, and subspecialty differences. However, current studies on training transfer have mainly focused on general continuing education, critical care nursing, or management training (12, 13), while research on neurosurgical specialty nurses within this complex specialty context remains limited. Existing studies have also rarely examined, from multiple perspectives including trainees, instructors, and training managers, the real process and mechanisms through which training outcomes are translated into clinical practice.

Therefore, this study adopted a descriptive qualitative design and conducted semi-structured interviews with neurosurgical specialty nurses, instructors, and training managers. The aim was to explore the practical manifestations and influencing mechanisms of training transfer among neurosurgical specialty nurses, thereby providing a reference for optimizing specialty nurse training design, improving post-training support systems, and promoting the effective translation of training outcomes into clinical practice.

## 2 Methods

### 2.1 Study design

This study adopted a pragmatic descriptive qualitative design to explore the characteristics and influencing factors of training transfer among neurosurgical specialty nurses through semi-structured interviews. Participants included trainees, instructors, and training managers, allowing the study to capture different perspectives on the training transfer process from training recipients, implementers, and organizational managers. This study was reported in accordance with the Consolidated Criteria for Reporting Qualitative Research (COREQ). Ethical approval was obtained from the ethics committee of the authors’ hospital, with the approval number 2025-0035-01. The interviews were conducted by two researchers who had received training in qualitative research and were carried out under the guidance of nursing experts experienced in qualitative research. There was no direct managerial or interest-related relationship between the researchers and the participants, thereby reducing potential bias during data collection.

### 2.2 Participants

From April to June 2025, purposive sampling was used to recruit individuals in Jiangsu Province who had participated in or were responsible for neurosurgical specialty nurse training programs. To enhance the richness and diversity of the data, variations in participants’ sex, age, professional title, years of clinical experience, hospital level, and regional background were considered during recruitment.

The inclusion criteria for trainees were as follows: registered nurses currently employed in clinical practice who had completed neurosurgical specialty nurse training and obtained a completion certificate; and those who provided informed consent and volunteered to participate in the study. The inclusion criteria for instructors were as follows: having undertaken theoretical or clinical practice teaching tasks in neurosurgical specialty nurse training programs; having worked in the field of neurosurgical nursing or a related area for at least 8 years; holding a bachelor’s degree or above and an intermediate or higher professional title; and providing informed consent and voluntarily participating in the study. The inclusion criteria for training managers were as follows: having participated in or been responsible for the organization, coordination, management, or evaluation of neurosurgical specialty nurse training programs; having worked in neurosurgical nursing or training management-related fields for at least 10 years; holding a bachelor’s degree or above and an intermediate or higher professional title; and providing informed consent and voluntarily participating in the study.

Participants were excluded if they were not on duty during the study period due to sick leave, maternity leave, resignation, or other reasons, or if they withdrew during the interview. Data collection and analysis were conducted concurrently. When no new concepts, categories, or themes emerged in consecutive interviews, data saturation was considered to have been reached, and recruitment was discontinued.

### 2.3 Data collection

The interview guide was developed based on Baldwin and Ford’s training transfer model and the FITT framework, and was revised in accordance with the specialty characteristics of neurosurgical nursing. The three interview guides shared a consistent overall structure and mainly focused on the current status and characteristics of training transfer, individual factors, training design factors, and organizational environmental factors. The interview focus was adjusted according to participants’ roles. Interviews with trainees focused on their experiences of applying knowledge and skills in neurosurgical nursing after training; interviews with instructors focused on teaching observations, curriculum design, and differences among trainees; and interviews with training managers focused on training organization, institutional support, resource allocation, and training evaluation. The detailed semi-structured interview guides for the three groups are provided in Supplementary Material 1.

Before the interviews, all participants received an explanation of the study and signed written informed consent forms. The researchers explained the study purpose, interview content, audio-recording procedures, confidentiality principles, and participants’ right to withdraw from the study. One-to-one semi-structured interviews were conducted at a time and place convenient for participants, mainly in quiet and private offices, departmental teaching rooms, or duty rooms. Telephone interviews were conducted for participants who were limited by geographical distance or work arrangements. During telephone interviews, the researcher conducted the interview in an independent space free from disturbance. Each interview lasted 30–60 min and was audio-recorded with the participant’s consent. One researcher led the interview, while another recorded the interview context, non-verbal information, and key content. Within 24 h after each interview, the audio recordings were transcribed verbatim and checked against the field notes. All transcripts were anonymized using participant codes, and any identifiable personal or institutional information was removed.

### 2.4 Data analysis

Framework analysis was used to analyze the interview data. After the interview transcripts were imported into a data management file, the researchers repeatedly read the transcripts to become familiar with the data. The research team developed an initial thematic framework based on the study aims, theories related to training transfer, and preliminary reading of the data, and then coded the transcripts. Content consistent with the initial framework was coded deductively, while newly emerging concepts from the data were coded inductively. The coded data were organized into an analytical matrix to compare similarities and differences in the perspectives of trainees, instructors, and training managers under the same themes. Through repeated discussions, the research team integrated, revised, and named the themes, and further interpreted the key factors influencing training transfer among neurosurgical specialty nurses and their interrelationships.

To enhance the reliability of the analysis, two researchers independently coded selected interview transcripts and resolved discrepancies through discussion. Throughout the research process, data from different sources were continuously compared, and field notes and researchers’ reflexive journals were used to verify the analytical findings, thereby enhancing the credibility and confirmability of the study.

## 3 Results

### 3.1 Participant characteristics

A total of 36 potential participants were approached in this study. Of these, two declined to participate because of scheduling conflicts, one was unable to be interviewed due to physical discomfort, and one did not respond to the invitation. By the 29th interview, the views and information obtained had become repetitive, and no new themes emerged. After three additional interviews were conducted without yielding new research information, data saturation was considered to have been reached, and recruitment was discontinued. The interviews lasted 35–62 min, with an average duration of approximately 48 min. Ultimately, 32 participants from 16 medical institutions of different levels in China were included, comprising tertiary grade A general hospitals, tertiary general hospitals, and secondary grade A general hospitals. The participants included 15 trainees, 11 instructors, and 6 training managers, all of whom held bachelor’s degrees.

The trainees mainly came from neurosurgery departments, neurological intensive care units, and neurosurgical intensive care units. Most were from tertiary grade A general hospitals (*n* = 10), while participants from municipal- and county-level hospitals were also included, providing a certain degree of representativeness. Among them, 12 trainees voluntarily applied to participate in the training, whereas 3 were nominated by their departments. All trainees had completed neurosurgical specialty nurse training and had returned to their posts to engage in neurosurgery-related clinical nursing work.

The instructors were nursing teachers or course leaders who undertook teaching responsibilities at provincial neurosurgical specialty nurse training bases. They were mainly involved in theoretical teaching, clinical practice supervision, and teaching management for specialty nurse training. Their teaching experience in specialty nurse education ranged from 2 to 7 years, and all had extensive experience in neurosurgical specialty nursing education and clinical teaching.

The training managers all had experience in the organization, coordination, teaching management, or quality evaluation of neurosurgical specialty nurse training. Their roles mainly included mentors, teaching secretaries who also served as academic coordinators, and teaching supervision experts. Most of them undertook multiple training management responsibilities while also holding nursing management positions in their departments. The managers mainly came from neurosurgery departments in tertiary hospitals. Their total neurosurgical experience ranged from 12 to 20 years, and their experience in training management or administrative coordination roles ranged from 4 to 12 years, indicating substantial expertise in both clinical practice and specialty nurse training management. Detailed characteristics of the participants are presented in Table 1.

**Table 1:** Demographic characteristics of interview participants (*N* = 32).

Characteristics/Items	Training graduates (*n* = 15)	Clinical instructors (*n* = 11)	Training administrators (*n* = 6)
Sex			
Female	13(86.67)	10(90.91)	6(100.0)
Male	2(13.33)	1(9.09)	
Age	34.93 ± 3.97(28 ~ 41)	37.55 ± 3.24(33 ~ 43)	39.67 ± 1.51(38 ~ 42)
Years of neurosurgical experience	11.53 ± 4.50(5 ~ 21)	12.27 ± 3.85(8 ~ 19)	15.83 ± 3.06(12 ~ 20)
Professional title			
Nurse practitioner	2(13.33)	0(0.00)	0(0.00)
Nurse-in-charge	12(80.00)	10(90.91)	4(66.67)
Associate chief nurse	1(6.67)	1(9.09)	2(33.33)
Administrative or management position			
Clinical nurse	4(26.67)		0(0.00)
Nursing team leader	11(73.33)	10(90.91)	2(33.33)
Ward head nurse	0(0.00)	1(9.09)	4(66.67)
Holding specialist nurse certification			
Yes	15(100.0)	5(45.45)	4(66.67)
No		6(54.55)	2(33.33)
Years holding specialist nurse certification	2.80 ± 1.61(2 ~ 8)		

Data are presented as mean ± standard deviation (range) or number (percentage),as appropriate.

### 3.2 Interview themes

A total of two themes and eight subthemes were identified: (1) practical manifestations of training transfer among neurosurgical specialty nurses; and (2) mechanisms influencing training transfer among neurosurgical specialty nurses. The first theme comprised three subthemes, describing the specific ways in which training outcomes were translated into clinical care and professional roles. The second theme was reorganized into two dimensions: facilitating factors and constraining factors influencing training transfer. Facilitating factors included individual motivation and professional identity, contextualized teaching, and organizational empowerment. Constraining factors included resource and sustainability limitations. This structure was developed to distinguish conditions that promote successful transfer from contextual barriers that restrict the implementation of acquired competencies.

**Figure 1:**
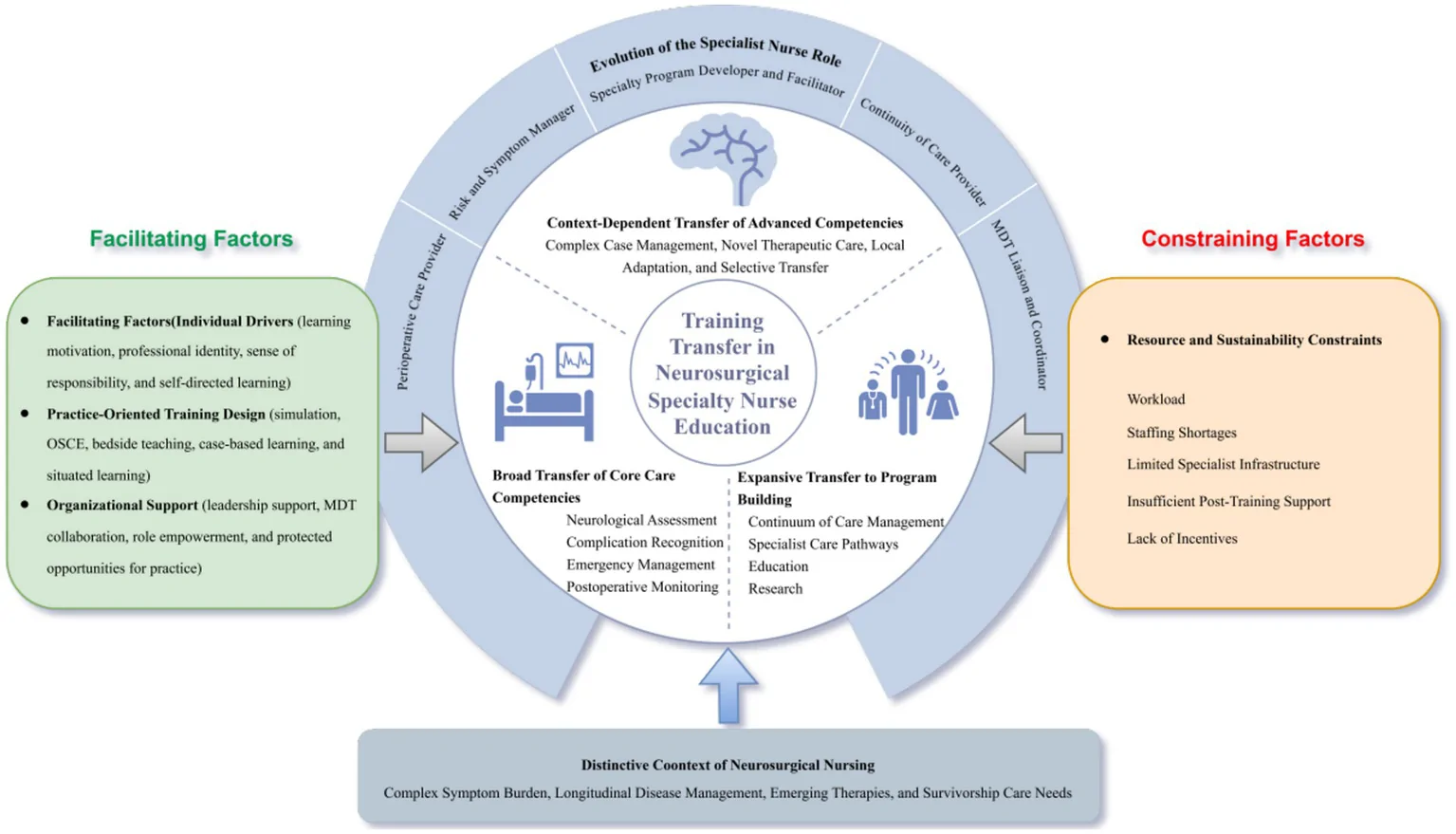
Conceptual framework of training transfer in neurosurgical specialty nurse education.

Based on these findings, this study further developed a conceptual framework of training transfer among neurosurgical specialty nurses (Figure 1), which systematically illustrates the dynamic relationships among the practical manifestations of training transfer, underlying mechanisms, distinctive contextual features of neurosurgical care, and the evolution of specialty nurse roles. The themes and subthemes are described below with representative quotations.

To protect participants’ privacy, all quotations were anonymized using participant codes: trainees were coded as S1–S15, instructors as T1–T11, and training managers as M1–M6. Additional supporting quotations are provided in Supplementary Material 2.

Theme 1: Practical manifestations of training transfer among neurosurgical specialty nurses.

#### 3.2.1 Relatively sufficient transfer of basic perioperative care content

After returning to their clinical posts, specialty nurses most smoothly transferred basic perioperative care content, including neurological assessment, observation of consciousness and pupils, vital signs monitoring, identification of postoperative complications, and tube care. These contents were closely related to the “daily nursing work of neurosurgical patients and were frequently used in clinical practice” (T11), making them easier to rapidly translate into routine nursing practice. Trainees reported that, after training, they were able to integrate multiple assessment indicators more systematically to judge postoperative changes in patients’ conditions, became “more sensitive to early signs of cerebral herniation” (S13), and shifted from “completing routine tasks” to “actively warning about related risks” (S2).

Case-based teaching and scenario simulation further strengthened trainees’ mastery of emergency identification and management procedures, enabling them to quickly draw on training experience and take appropriate actions when encountering similar situations after returning to work. As one trainee stated, “The OSCE station simulated a patient with a temporal lobe tumor who suddenly developed status epilepticus. After returning to work, I actually encountered a similar situation, and I was immediately able to follow the airway protection procedure that had been practiced during the simulation” (S8). Managers also observed that improvement in these basic competencies was directly reflected in nursing quality and patient safety management: “After training, specialty nurses became more sensitive to changes in the conditions of neurosurgical patients, and postoperative observation and risk warning in the department also became more standardized” (M1).

#### 3.2.2 Limited transfer of advanced techniques and complex case management

Compared with basic care content, the implementation of advanced competencies, including complex case management, advanced monitoring techniques, and precision treatment-related nursing, was more dependent on institutional readiness and available practice environments. Such content was highly dependent on the hospital’s case mix, equipment conditions, and foundation for multidisciplinary collaboration. After returning to their original posts, some specialty nurses encountered a mismatch between the advanced competencies acquired during training and the clinical resources available within their institutions. Consequently, some competencies could not be fully implemented because of limited case exposure, insufficient technical platforms, or immature multidisciplinary systems rather than deficiencies in training itself. As participants noted, some advanced content could only “remain at the level of knowledge understanding” (T5) or serve as “conceptual updating” (M2), while complex skull base tumor care procedures and nursing techniques related to emerging treatments were difficult to sustain in routine practice (S14).

Although some trainees found it difficult to fully replicate the training content, they still engaged in selective transfer and local adaptation according to the conditions of their own hospitals. As one trainee stated, “In real application, it may not be possible to bring everything back exactly as it was taught, but I will adjust it according to the actual situation in my department” (S10). Some trainees adapted ideas, observation approaches, or management processes learned during training and applied them to existing patient management, such as optimizing temperature management procedures (S4) and adjusting the focus of cerebral edema observation (S5). Teacher T3 and manager M3 also considered this reprocessing and local adaptation based on clinical context to be an important manifestation of training transfer.

#### 3.2.3 Extension of training outcomes from individual care to specialty development

As training content was gradually internalized, some trainees’ roles began to extend from direct clinical care to teaching, quality control, and specialty development. In addition to direct clinical nursing, some trainees gradually undertook departmental training, specialty process optimization, and quality improvement after returning to work. For example, one trainee reported, “I adapted the OSCE case on intracranial pressure crisis management from the training into teaching material for our department” (S7), while another stated, “After the training, I participated in revising the health education process for patients with brain tumors in our department” (S9). These activities promoted the diffusion of training outcomes from the individual level to the team and organizational levels.

The training also enhanced trainees’ problem awareness and evidence-based thinking. Some trainees began to actively identify clinical challenges in neurosurgical nursing and attempted to promote practice improvement through research or quality improvement projects. As one trainee stated, “I used to feel that research was far away from me. After the training, I began to actively think about what problems in neurosurgical specialty nursing could be improved or developed into research topics” (S6). Managers believed that this role evolution indicated that training transfer had gone beyond individual skill acquisition and “played a driving role in improving the quality of specialty nursing in the department” (M4), thereby promoting specialty capacity building and disciplinary development within the department.

*Theme 2*: Facilitators and barriers influencing training transfer among neurosurgical specialty nurses.

#### 3.2.4 Facilitator 1: individual motivation and professional identity

Clear learning goals, specialty development needs, and professional identity were important intrinsic drivers of training transfer. Trainees’ main motivations for participating in training included actively pursuing specialty development, improving specialty nursing competence, meeting departmental development needs, and obtaining career development opportunities (S2). Trainees who enrolled voluntarily and had a strong intention for specialty development showed higher engagement during training and were more likely to actively apply and promote what they had learned after returning to work. As one teacher noted, “Trainees who come to learn actively and with departmental problems in mind ask more questions in class, and after returning to work, they are more willing to try to apply the new concepts and methods they encountered during training” (T8).

Some trainees mentioned that the training not only improved their professional competence but also strengthened their understanding of and awareness of their responsibility for patient care. One trainee stated, “Patients with severe neurosurgical conditions and their families are under great psychological pressure. After the training, I felt that nurses should not only carry out treatments but also play a role in communication and support” (S1). Meanwhile, training content related to psychological support and palliative care deepened trainees’ understanding of the specialty nurse role, shifting their focus from simply monitoring postoperative vital signs to “paying more attention to patients’ anxiety, functional recovery, and quality of life after discharge” (S13). This enhanced professional identity further consolidated their willingness to continue applying what they had learned.

#### 3.2.5 Facilitator 2: contextualized and practice-oriented teaching

The degree to which training content was aligned with real clinical scenarios directly influenced training transfer. The closer the training content was to authentic neurosurgical nursing scenarios, the smoother its translation after trainees returned to work. For trainees from hospitals with well-developed neurosurgical subspecialties, the disease-specific content across different subspecialties was highly consistent with departmental needs: “The training content was very compatible with the needs of our department. Postoperative observation, complication identification, and rehabilitation guidance are used every day” (S8). As a result, they were able to quickly integrate what they had learned into daily practice. However, when the curriculum exceeded the hospital’s current scope of diagnosis and treatment or when the case mix differed substantially, “even if this part of the content was understood, there was basically no opportunity to use it after returning,” and transfer was significantly limited (S14).

Teachers believed that curriculum design should balance general applicability with specialty-specific characteristics. As one teacher stated, “Brain tumor nursing cannot only cover general neurosurgical content; it also needs to be combined with nursing priorities after different tumor types and different surgical approaches” (T7). A needs-oriented tiered training system should therefore be established: “A needs assessment should be conducted before training, and targeted teaching should be provided at different levels where appropriate” (T9).

Participants consistently believed that practice-oriented teaching methods, such as case analysis, scenario simulation, and bedside teaching, were more helpful for trainees in establishing links among “knowledge, context, and action,” thereby reducing the difficulty of applying what they had learned after returning to work. One trainee stated, “The teacher guided us at the bedside to analyze real cases and observe postoperative changes in patients. This left a much deeper impression than simply listening to lectures and made it clearer what we should observe and how we should manage similar situations after returning to work” (S3). Another trainee noted, “The simulation of sudden postoperative seizures or changes in consciousness in neurosurgical patients was very helpful for handling similar situations after returning to work. When I actually encountered such situations, I did not panic as much” (S6).

Especially in neurosurgical emergency management, “a complete simulation of the process from detecting abnormal changes in a patient’s condition to reporting, management, and transfer” (T6) could significantly improve trainees’ clinical decision-making and emergency response abilities. Teachers also pointed out that assessment should focus on problem identification and comprehensive decision-making in real clinical contexts. Compared with simple theoretical tests, “OSCEs and nursing care plan design are better able to reflect trainees’ integrated application of neurosurgical specialty knowledge” (T10). Managers further suggested that new technologies such as virtual reality may provide a direction for immersive teaching, although their application is currently limited by equipment conditions (M1).

#### 3.2.6 Facilitator 3: organizational empowerment and team collaboration

Leadership support, clearly defined role responsibilities, and mature multidisciplinary collaboration mechanisms were important organizational safeguards for training transfer. When hospitals clarified the roles and responsibilities of specialty nurses and granted them practical authority, training outcomes were more likely to be transformed into stable specialty practice. One trainee stated, “My head nurse was very supportive. After I returned to the department, she asked me to be responsible for health education for neurosurgical patients and part of the quality control work, so I had opportunities to use what I had learned” (S5). Another trainee noted, “The nursing department established a specialty nurse management system that requires regular summaries and outcome reports, covering clinical practice, teaching, research, and other aspects. This motivated me to apply what I had learned in clinical practice” (S7). In contrast, “if the department does not give specialty nurses a clear role, they are still busy with routine work after returning, and much of the training content is difficult to truly implement” (T9).

The care of neurosurgical patients involves not only neurosurgery but also rehabilitation, nutrition, psychology, and other disciplines, and therefore “requires support from doctors and other teams in multiple aspects” (S11). “Whether the team is willing to cooperate directly affects whether specialty nurses can put the training content into practice” (T2). A supportive team climate and peer support could reduce resistance to translating training content: “When carrying out the project, leaders and colleagues were very cooperative and tried to create conditions for me to implement new skills and methods” (S9), thereby facilitating work progress.

Conversely, if clear role positioning or multidisciplinary platforms were lacking, the roles assigned to trainees during training were difficult to maintain after they returned to work, even if they had received relevant training. One manager explained, “If the hospital does not have a disease-specific management model or clearly define the specialty nurse’s role as an MDT coordinator, then the navigation, follow-up, coordination, and communication skills learned during training will be difficult to put into play” (M6).

#### 3.2.7 Barrier 1: organizational resource mismatch and insufficient post-training support

Insufficient physical resources, lack of protected time, absence of performance incentives, and discontinuity in post-training support jointly constituted practical constraints on the sustainability of training transfer. As one trainee stated, “Some content requires equipment or information system support, but the current conditions in our department are limited” (S12), allowing only simple improvements to be made. Heavy clinical workload also made it difficult to promote specialty projects: “Most of the time, we can only complete routine treatment and nursing care first. We want to carry out specialty projects, but there is not enough time” (S15).

Some trainees pointed out that the lack of independent subspecialty monitoring units or palliative care teams in their hospitals meant that “all patients are scattered across general neurosurgical wards” (S10), making it difficult to truly implement training content related to refined disease-specific management (S10) and end-of-life communication (S1). In terms of incentives, the professional contributions of specialty nurses were not fully reflected in performance distribution or promotion pathways. One trainee stated, “After returning, I had more tasks, including teaching, quality control, and research, but there was no obvious change in performance rewards or recognition” (S9), which weakened motivation for sustained application. Without “dedicated posts for neurosurgical specialty nurses, differentiated performance incentives, and career development pathways” (T1), the long-term benefits of training investment would be weakened, nurses could become prone to burnout, and the stable transformation of training outcomes would be affected.

In addition, the depth of continuous guidance and communication decreased substantially after training ended. One trainee reported, “During the training, my mentor communicated with me and provided guidance every week. But after returning to the hospital, there was almost no one to discuss problems with” (S3). Managers also observed that “after trainees return to their original institutions, they lack guidance from specialty clinical mentors, and the training effect gradually weakens after a few months” (M5). Participants generally expressed the hope that “a deeper learning and communication platform and continuous practice guidance could be established” (S4) to enhance the stable translation of training outcomes.

Managers also acknowledged that current training places more emphasis on short-term learning outcomes. Although post-training support such as online learning platforms and annual conference exchanges has played a certain role, “systematic follow-up, feedback, and continuous guidance are still insufficient, especially regarding the ongoing promotion of practice projects after trainees return to their posts” (M4). This, to some extent, affected the long-term maintenance of training transfer.

#### 3.2.8 Contextual driver: neurosurgical complexity and specialty role expansion

Neurosurgical patients often experience complex and rapidly changing conditions, requiring continuous monitoring, rehabilitation support, and coordinated long-term care. The role of specialty nurses has therefore expanded beyond perioperative care toward broader responsibilities involving patient education, rehabilitation guidance, care coordination, and follow-up management (14, 15). These continuing care needs have promoted the transformation of specialty nurses from ward-based care providers to full-cycle managers, continuing care coordinators, and subspecialty liaisons (15, 16). As one trainee stated, “I used to think that it was enough to provide good nursing care during hospitalization. Now I also consider how patients will recover after returning home and how their family members will provide care” (S12).

A few trainees also mentioned that, in actual practice, they had gradually begun to undertake patient coordination and specialty liaison roles, “serving as a bridge in communication among neurosurgery, critical care teams, and rehabilitation teams” (S11). Meanwhile, the rapid development of minimally invasive neurosurgical techniques, neuronavigation, neuromodulation, and intraoperative neuromonitoring further increased specialty nurses’ need for lifelong learning and competency updating. One trainee stated, “New technologies in neurosurgery are developing rapidly now, and nurses also need to understand related observation and health education” (S1). Another noted, “The training made me realize that I cannot rely only on previous experience. Neurosurgical specialty nursing knowledge is updated quickly, and continuous learning is necessary” (S3).

Teachers also believed that “the future development direction of neurosurgical specialty nurses should be full-cycle management covering postoperative observation, rehabilitation, health education, follow-up, and psychological support” (T3). This role expansion is both a consequence of disease complexity and an inevitable pathway for deepening the value of specialty nursing.

## 4 Discussion

This study explored the characteristics and influencing factors of training transfer among neurosurgical specialty nurses from the perspectives of trainees, instructors, and training managers. The findings indicate that training transfer among neurosurgical specialty nurses is not a simple replication of training knowledge or skills into clinical practice, but rather a contextualized process dynamically shaped by individual readiness, training design, organizational support, and the distinctive clinical characteristics of neurosurgical specialty care (17, 18). Basic competencies, such as neurological assessment, perioperative observation, complication identification, and patient education, were more readily transferred because of their high relevance to daily clinical practice. In contrast, advanced competencies, including complex case management, neurocritical care techniques, specialty treatment-related nursing, and multidisciplinary collaboration, were more dependent on case resources, institutional conditions, technical platforms, and team support (19, 20). These findings suggest that training transfer among neurosurgical specialty nurses should be understood as an adaptive and contextualized process rather than a linear application of training content.

### 4.1 Contextual dependence of training transfer among neurosurgical specialty nurses

This study found that training content highly aligned with daily clinical work was more likely to be applied after specialty nurses returned to their posts. Beyond confirming the influence of learner characteristics, training design, and work environment described in classical training transfer models, our conceptual framework further extends existing models by incorporating specialty-specific contextual conditions of neurosurgical nursing. Specifically, Figure 1 illustrates that training transfer is not only determined by individual readiness, instructional design, and organizational support, but also shaped by the availability of clinical scenarios, subspecialty resources, multidisciplinary collaboration, and evolving specialty nurse roles. This framework highlights an adaptive reconstruction process in which specialty nurses selectively integrate and modify acquired competencies according to their local clinical environments rather than simply reproducing standardized training content. Neurosurgical patients commonly experience rapid postoperative changes, increased intracranial pressure, neurological deficits, cognitive impairment, dysphagia, and psychological distress (21). Therefore, training content related to patient safety, early warning, perioperative monitoring, and risk identification can be directly integrated into routine nursing practice.

However, the transfer of advanced training content was relatively limited. Although trainees were exposed during training to complex postoperative management, neurocritical care, precision treatment-related nursing, rehabilitation, and follow-up care, whether these competencies could be applied after returning to work largely depended on the clinical environment of their original institutions. Hospitals with limited subspecialty case volume, insufficient equipment, or immature multidisciplinary team mechanisms provided relatively fewer practice opportunities for advanced content transfer (22, 23).

It is worth noting that the inability to directly apply advanced content does not necessarily indicate training failure. Some trainees transformed advanced training concepts according to the actual conditions of their institutions, such as adjusting the frequency of condition monitoring, improving patient education content, revising nursing procedures, or developing departmental training materials. This suggests that training transfer among neurosurgical specialty nurses involves a process of “adaptive reconstruction,” in which trainees selectively transform training content according to available resources and clinical needs.

### 4.2 Individual factors influencing training transfer

Learning motivation, previous clinical experience, and proactive transfer awareness were important individual factors influencing training transfer. Domestic studies on specialty nurse training transfer have also indicated that individual motivation to transfer, opportunities for workplace application, organizational support, and the fit between training content and clinical practice all affect the translation of training outcomes into clinical practice (24, 25).

Trainees with clear learning goals and strong needs for professional development were more likely to connect training content with clinical problems and actively apply what they had learned after returning to work (26, 27). Previous experience also influenced the depth of training transfer. Nurses with extensive neurosurgical experience, especially subspecialty care experience, were more likely to integrate training content with real clinical cases and further translate it into practice. In contrast, trainees from hospitals with fewer subspecialty cases or a weaker specialty foundation may face greater difficulties in understanding and applying complex training content.

These findings suggest that training design should fully consider trainees’ baseline competencies and institutional backgrounds. A uniform training model may not meet the needs of nurses working in different clinical contexts. Tiered training based on clinical experience, case exposure, and expected post-training roles may help improve the relevance of training content and the effectiveness of training transfer.

### 4.3 Implications for training design

The findings of this study suggest that neurosurgical specialty nurse training should shift from simple knowledge transmission to the cultivation of contextualized application competencies. Before the training program begins, a needs assessment should be conducted, taking into account hospital level, case mix, available resources, trainees’ baseline competencies, and their expected specialty nurse roles after returning to work.

A tiered curriculum may be more suitable for neurosurgical specialty nurse training. Core content, such as neurological assessment, perioperative monitoring, complication identification, seizure management, intracranial pressure-related observation, rehabilitation guidance, and patient education, should be included as basic training content for all trainees. Advanced content, such as precision treatment-related nursing, complex case management, neurocritical care techniques, MDT coordination, follow-up management, and research translation, may be more appropriate for trainees from institutions with mature specialty platforms or corresponding clinical needs.

In addition, case-based teaching, simulation training, bedside teaching, workshops, and mentorship should be further strengthened. Given the rapid postoperative changes and high risks among neurosurgical patients, simulation training focused on scenarios such as cerebral herniation, seizures, acute deterioration of consciousness, postoperative bleeding, and cerebrospinal fluid leakage may help improve trainees’ clinical judgment and emergency response capabilities.

Training evaluation should also not stop at completion assessments. Although theoretical and skills examinations are useful for evaluating knowledge acquisition, they are insufficient for reflecting training transfer in real work settings. Future training programs should include indicators such as post-return follow-up, post-training practice projects, quality improvement outcomes, specialty teaching activities, and changes in clinical practice to more comprehensively evaluate training transfer.

### 4.4 Organizational support and role development

Training transfer is also influenced by organizational conditions. Leadership support, clear role positioning, multidisciplinary collaboration, resource accessibility, workload, performance recognition, and career development opportunities all affect whether trainees can apply and sustain what they have learned (28). Explicit authorization and role positioning are particularly important. If specialty nurses lack clearly defined responsibilities after returning to work, or if they have no opportunity to participate in neurosurgical subspecialty management, training outcomes may remain only at the level of individual knowledge. Conversely, when institutions assign specialty nurses responsibilities in patient education, quality improvement, teaching, follow-up management, or MDT coordination, training transfer is more likely to be sustained.

Neurosurgical nursing is inherently multidisciplinary, involving neurosurgery, neurology, critical care medicine, rehabilitation, nutrition, psychology, and palliative care. Therefore, specialty nurses need not only clinical knowledge but also opportunities to participate in collaborative care. Healthcare institutions should clarify the roles of neurosurgical specialty nurses in MDTs, patient navigation, symptom management, and long-term follow-up.

In addition, workload and resource constraints need to be addressed. Specialty nursing activities, including symptom assessment, follow-up, health education, quality improvement, and research, require time, human resources, and institutional support. Without protected time and reasonable incentives, even highly motivated trainees may find it difficult to sustain training transfer. Therefore, performance evaluation and career development mechanisms should recognize both the visible and less visible contributions of specialty nurses in complex neurosurgical care.

### 4.5 Practical implications in the specialty context

Compared with general nursing training, training transfer among neurosurgical specialty nurses shows more pronounced specialty-specific contextual characteristics. Differences in disease types, surgical approaches, disease stages, and treatment modalities influence the applicability of training content. In addition, postoperative emergencies, rapidly evolving treatment technologies, and long-term rehabilitation and psychosocial care needs further increase the complexity of training transfer.

These findings suggest that future training programs should include both common and complex neurosurgical cases and regularly update curriculum content according to developments in minimally invasive techniques, neuronavigation, neuromodulation, intraoperative monitoring, and neurorehabilitation. At the same time, full-cycle management training should be strengthened so that specialty nurses are competent not only in perioperative nursing but also in symptom management, psychological support, family education, rehabilitation guidance, and long-term follow-up.

**Figure 2:**
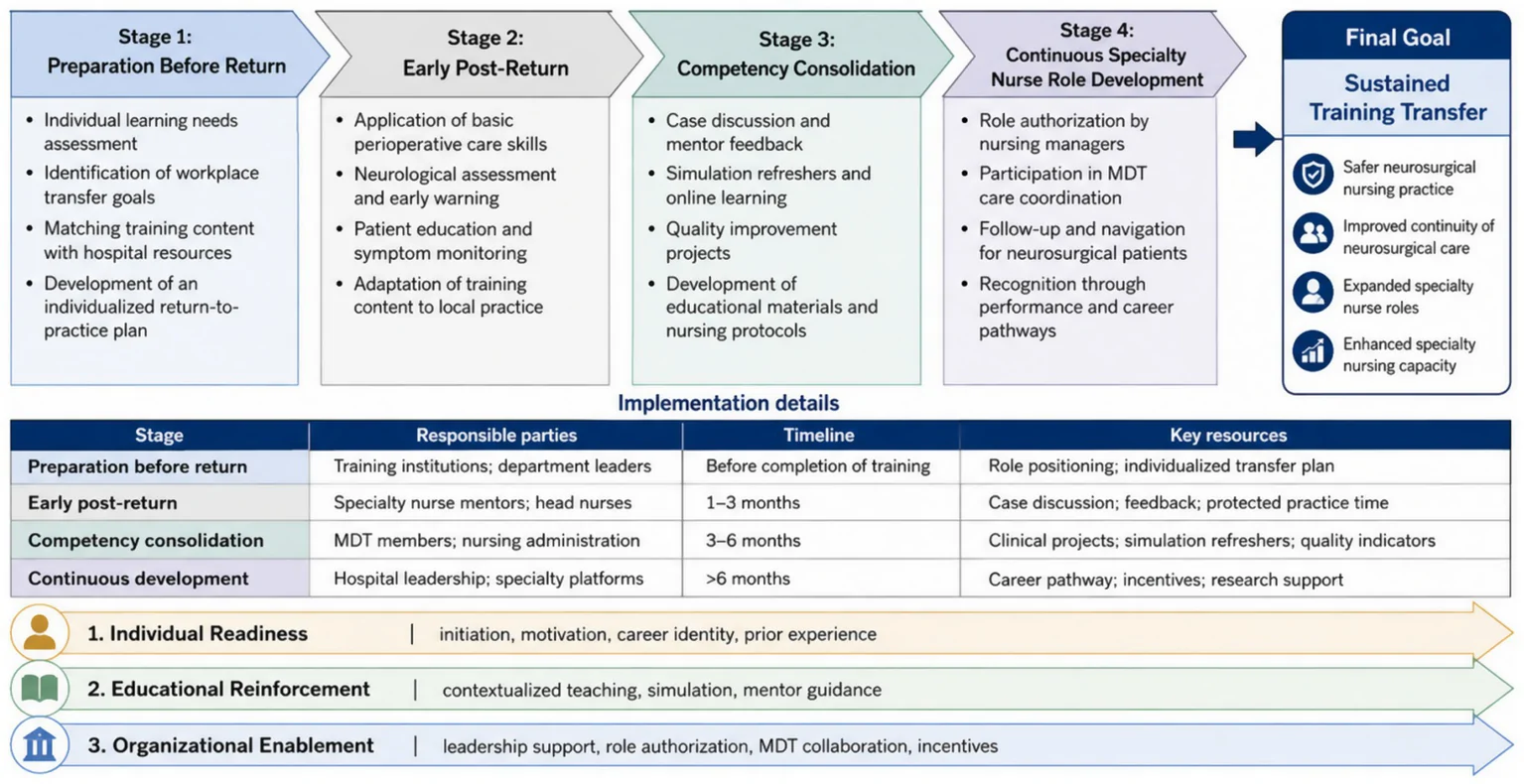
Post-training support pathway for sustaining training transfer among neurosurgical specialty nurses.

Based on the findings of this study, we developed a post-training support pathway (Figure 2) derived directly from participants’ descriptions of facilitators and barriers affecting training transfer. The revised pathway specifies responsible stakeholders, implementation timelines, and required resources across four stages: pre-return preparation (training institution and department leaders; within 1 month before completion), early post-return reinforcement (specialty nurse mentors and clinical managers; within 1–3 months after return), competency consolidation (department teams and multidisciplinary collaborators; 3–6 months after return), and continuous role expansion (hospital nursing administration and specialty platforms; beyond 6 months). The proposed pathway is therefore not an additional intervention model independent of the qualitative findings, but a practical translation of the mechanisms identified from interviews. This pathway embeds structured support at four key time points: preparation before returning to work, early post-return, competency consolidation, and continuous role expansion. At each stage, individual readiness, educational reinforcement, and organizational support are parallel prerequisites rather than sequential steps. For example, without role authorization and protected practice time, even highly motivated nurses may find it difficult to apply advanced competencies; without simulation refresher training and mentor feedback, early training gains may diminish within several months. This integrated pathway is consistent with the Baldwin–Ford model and addresses trainee characteristics, training design, and work environment, providing a practical template for maximizing the return on investment in specialty nurse education.

## 5 Strengths and limitations

This study has several strengths. First, it included trainees, instructors, and training managers, enabling a multi-perspective understanding of the training transfer process among neurosurgical specialty nurses. Second, this study focused on a highly specialized and complex clinical context, revealing how specialty-specific contextual factors influence training transfer. Third, framework analysis was used, allowing a balance between theoretical guidance and inductive interpretation of the data, which facilitated a systematic explanation of the findings.

However, this study also has several limitations. First, as a qualitative study, it aimed to provide an in-depth understanding rather than statistical generalization. Second, although trainees, instructors, and training managers were included, physicians, rehabilitation therapists, psychological professionals, patients, and family caregivers were not involved. Given the multidisciplinary nature of neurosurgical nursing, future studies could integrate the perspectives of these groups to gain a more comprehensive understanding of training transfer and its impact on team collaboration and patient care. Third, the data were mainly derived from participants’ retrospective accounts, which may be subject to recall bias and social desirability bias. Future mixed-methods studies could combine interview data with objective indicators, such as quality improvement outcomes, changes in clinical practice, patient outcomes, and completion of post-return practice projects. Finally, this study did not adopt a longitudinal design; therefore, it was unable to capture how training transfer is maintained, weakened, or deepened over time. Future longitudinal studies are needed to examine the trajectories of training transfer among nurses at different stages after returning to their clinical posts.

Neurosurgical patients often present with complex and rapidly changing conditions, and the quality of specialty nursing care is highly dependent on nurses’ clinical judgment, risk identification, and continuous care competencies. This study indicates that the value of specialty nurse training lies not only in the acquisition of knowledge and skills, but also in whether training content can be effectively integrated into real work contexts and continuously applied in clinical practice. Therefore, continuing education for neurosurgical specialty nurses should place greater emphasis on the contextual fit between training content and job requirements, and should strengthen practice-based and workplace-oriented teaching design.

This study further suggests that training transfer requires sustained support from the organizational environment. Healthcare institutions should provide specialty nurses with clearly defined job responsibilities, opportunities for multidisciplinary collaboration, protected practice time, and continuous development pathways. Through post-training guidance, post-return evaluation, and professional support networks, institutions can promote the sustained transformation of training outcomes into patient education, symptom management, rehabilitation guidance, quality improvement, and specialty service capacity. These strategies may help improve the level of neurosurgical specialty nursing practice and further enhance the care experience and quality of care for patients and their families.

## 6 Conclusion

This study shows that training transfer among neurosurgical specialty nurses is a dynamic process jointly influenced by learner characteristics, training design, and the organizational environment. Basic and frequently used clinical care competencies, such as neurological assessment, perioperative observation, complication identification, and patient education, are more readily transferred. In contrast, advanced competencies, including complex case management, neurocritical care, interdisciplinary collaboration, and specialty project management, are more dependent on case resources, organizational support, practice opportunities, and continuous post-training reinforcement. The findings also indicate that training outcomes are reflected not only in improvements in direct clinical care competencies but may also extend to specialty teaching, quality improvement, research practice, and specialty management.

These findings suggest that training transfer among neurosurgical specialty nurses is not a linear application of training knowledge, but rather an adaptive process of integration and reconstruction within real work contexts. Future continuing education programs for neurosurgical specialty nurses should strengthen pre-training needs assessment, establish tiered, contextualized, and practice-oriented curricula, reinforce case-based teaching, simulation training, post-return practice, and continuous post-training support, and extend training evaluation to the actual effects of practice transformation in real clinical settings. At the organizational level, healthcare institutions should further clarify the role positioning of specialty nurses, promote their participation in multidisciplinary collaboration and specialty management, and provide necessary resource support, performance recognition, and career development opportunities to facilitate the sustained transfer of training outcomes and improve the quality of neurosurgical specialty nursing.

## Data Availability

The raw data supporting the conclusions of this article will be made available by the authors, without undue reservation.
