# Supplementary material for: Exploring training transfer in neurosurgical specialty nurse education: a qualitative descriptive study
Source: Front Neurol. 2026 Sep 11;17:1915268. doi: 10.3389/fneur.2026.1915268 (PMC13612333; doi:10.3389/fneur.2026.1915268)
Supplement: Supplementary file 1 [file Supplementary_file_1.DOCX]

**Supplementary information 1 : Interview Guide (online only)**

**Introduction and Opening Remarks (for all participants)**

Hello! Thank you for taking part in this interview. My name is [______], and I am a [position/role] from [XX University/XX Hospital]. I am currently conducting a qualitative study on the “characteristics and influencing factors of training transfer among neurosurgical specialist nurses.” This study aims to understand how neurosurgical specialist nurses apply the knowledge and skills they have learned during training to clinical practice, as well as the factors that may influence this process.

As hospitals, work roles, and personal experiences vary, each participant’s experiences and perspectives on training and clinical application are uniquely valuable. Therefore, we are interviewing specialist nurses, educators, and training administrators to gain a comprehensive and in-depth understanding of how neurosurgical specialist nurse training is translated into clinical practice.

This interview is expected to last approximately 40–60 minutes. Your participation is entirely voluntary. During the interview, you may pause or withdraw at any time, or choose not to answer any questions. Please be assured that this interview will not have any impact on you, and you are free to share your views based on your own experiences.

With your permission, I would like to audio-record this interview so that I can focus more fully on our conversation and accurately organize your views during the subsequent analysis. All recordings and interview data will be kept strictly confidential and used only for academic research. Only members of the research team will have access to them. In research reports or publications, your real name will be replaced with an anonymous code, and no information that could identify you will be disclosed.

May I ask whether you agree to the audio recording and to begin the interview?

**Semi-Structured Interview Guides for Three Participant Groups**

| **Trainees** | **Teachers** | **Training managers** |
| --- | --- | --- |
| **Current transfer of neurosurgical specialist nurse training into clinical practice**   - Could you briefly describe your experience of participating in the neurosurgical specialist nurse training programme? - After returning to your post, what training content have you applied when caring for neurosurgical patients? Could you describe this with reference to specific cases? - Which aspects of neurosurgical specialist nursing training have you used most frequently in clinical practice? Which aspects have you used less often, and why? - Since completing the training, what changes have occurred in the way you apply your specialist nursing competencies? What do you think these changes are related to? - Apart from direct clinical nursing care, have you applied the training content in other work? If so, how effective has this been?   **Individual factors influencing training transfer**   - What were your main reasons for participating in the neurosurgical specialist nurse training programme? - How did your motivation influence your learning and willingness to apply the training content after returning to work? - What differences existed between your previous neurosurgical nursing experience and the content delivered during training? - How did these differences influence your understanding, absorption and later application of the training content? - After completing the training, what was your attitude toward applying what you had learned? - Did you actively seek opportunities to use the training content? - When practical conditions were insufficient, how did you usually respond? - How did training content related to communication, psychological support or palliative care influence your professional identity and daily practice?   **Training design and educational support**   - How well did the training content match the actual needs of neurosurgical nursing practice? - Which contents were most consistent with clinical needs? - Which contents were relatively difficult to apply, and why? - With the development of new technologies in neurosurgery, such as minimally invasive techniques, neuronavigation and intraoperative monitoring, did the training help you develop the ability to continue learning and updating your knowledge? - Which teaching or assessment methods were most helpful for your later clinical application, and why? - After the training, what forms of continuing learning or professional support did you receive? - How did these supports contribute to your subsequent professional development?   **Organisational environment and specialty development**   - After returning to work, what was the attitude of your department or hospital toward your involvement in neurosurgical specialist nursing care? - What support did your department or hospital provide? - What additional support would you hope to receive in the future? - What organisational or environmental difficulties have you encountered when applying the training content? - How did you usually solve or respond to these difficulties? | **Observed transfer of neurosurgical specialist nurse training into practice**   - Could you briefly describe the teaching module you were responsible for in the neurosurgical specialist nurse training programme? - From your observation, what are the most obvious changes among trainees after they return to their clinical posts, particularly in relation to the care of neurosurgical patients? - Based on your teaching experience, which aspects of neurosurgical specialist nursing are most easily transferred into clinical practice? - Which aspects are more difficult to transfer, and why? - How do you usually learn about trainees’ application of training content after they return to work? - Could you share any memorable examples of post-training application?   **Observed individual differences among trainees**   - In your view, how do trainees with different learning motivations differ in their transfer of training? - How do trainees with different clinical backgrounds differ in their application of training content? - How does trainees’ previous experience in neurosurgical nursing influence their mastery and application of the training content? - How does the level or type of hospital in which trainees work affect their ability to apply what they have learned? - How have you observed these differences during or after training?   **Reflections on training design**   - In your view, how well does the current neurosurgical specialist nursing curriculum match clinical needs? - Which contents need further optimisation? - How do you accommodate differences in trainees’ hospital levels, clinical backgrounds and practical needs during teaching? - Which teaching methods are most helpful in promoting trainees’ post-training application? - What difficulties arise during the implementation of these teaching methods? - How do you view the current follow-up evaluation mechanism after training? - Is there insufficient continuing support after trainees return to their posts? - Are the current assessment and post-training support mechanisms sufficient to promote the transformation of training content into clinical practice? - How could these mechanisms be improved in the future?   **Organisational environment and specialty development**   - From your perspective, which organisational environmental factors most strongly influence trainees’ transfer of training after returning to work? - Which hospital-level institutional factors are most likely to affect the implementation of neurosurgical specialist nursing training outcomes? - In relation to the future development of neurosurgical specialist nurses, what aspects of the training system and support environment need further improvement? | **Overall implementation and transfer of the training programme**   - Could you briefly describe the overall operation of the neurosurgical specialist nurse training programme that you manage? - From a management perspective, what changes have occurred after training in terms of neurosurgical patient care quality, teaching atmosphere or specialty development? - How would you describe the current clinical transfer of neurosurgical specialist nursing training content? - Which contents are easier to implement in clinical practice? Which are relatively difficult, and why? - To what extent are trained specialist nurses currently able to undertake neurosurgical specialist nursing roles?   **Management observations of individual factors**   - In your view, do specialist nurses participating in the training have a clear professional role orientation? - How does professional role orientation influence the sustained application of training outcomes? - During trainee selection, do you consider applicants’ previous experience in neurosurgical nursing or the case structure of their departments? - What differences exist in training transfer among trainees from different hospital backgrounds? - How do individual motivation, prior experience and post responsibilities influence the clinical use of training outcomes?   **Training system design**   - How well does the current training content match the practical needs of different hospitals? - When designing the curriculum, how do you balance advanced knowledge with clinical practicality? - What teaching and evaluation methods are currently used in the training programme? - How effective are these methods in promoting clinical application? - How could these methods be improved in the future? - After the training, has a continuous follow-up, feedback or support mechanism been established? - What limitations remain in the current post-training support system?   **Organisational and institutional support**   - How does the hospital currently support specialist nurses in terms of post setting, performance incentives or career development? - What are the greatest organisational or resource barriers in the development of neurosurgical specialist nursing? - Does the multidisciplinary team collaboration mechanism support specialist nurses’ participation in the whole-course management of neurosurgical patients? - What problems still exist in multidisciplinary collaboration? - In the future, how should the training system and support mechanisms be optimised to better promote the transfer of neurosurgical specialist nursing training into clinical practice? |

**Concluding Remarks (for all participants)**

That brings us near the end of today’s interview. Thank you very much for taking the time to share your experiences and valuable insights.Finally, regarding the topic of “training transfer among neurosurgical specialist nurses,” is there anything else you would like to add, or anything important that you think we have not discussed?

Thank you again for your participation. If you think of any additional information later, please feel free to contact me at any time. I wish you all the best in your work.
