# Supplementary material for: Exploring training transfer in neurosurgical specialty nurse education: a qualitative descriptive study
Source: Front Neurol. 2026 Sep 11;17:1915268. doi: 10.3389/fneur.2026.1915268 (PMC13612333; doi:10.3389/fneur.2026.1915268)
Supplement: Supplementary file 2 [file Supplementary_file_2.DOCX]

**Supplementary Material 2. Representative quotations for themes and subthemes**

| **THEME ONE: Practical manifestations of training transfer among neurosurgical specialist nurses** | |
| --- | --- |
| **Sub-theme: Relatively sufficient transfer of basic perioperative care content** | *Basic assessments of neurosurgical patients, including consciousness, pupils, muscle strength, and intracranial pressure, as well as postoperative observation procedures and nursing ward rounds, were most closely connected with daily nursing work. Therefore, these components were applied most frequently after trainees returned to their posts, and changes were most readily observed.*  *T11*  *After the training, I was able to integrate vital signs, consciousness, pupils, muscle strength, drainage status, and other indicators more systematically when assessing postoperative patients. I also became more sensitive to some early signs of cerebral herniation.*  *S13*  *In the past, I mainly followed routine nursing procedures. Now I take a more proactive approach and think about whether the patient is at risk of increased intracranial pressure, seizures, or neurological changes.*  *S2* |
| **Sub-theme: Limited transfer of advanced techniques and complex case management** | *Some trainees came from centers with a large volume of complex neurosurgical cases, where many advanced specialist nursing techniques were already well established and could be applied soon after they returned. However, in some hospitals, case volumes were low, especially for complex skull base tumors and tumors in eloquent brain areas. Although trainees understood the content taught during the training, they lacked sufficient clinical scenarios and conditions to practise it after returning to work; consequently, the training content remained largely at the level of conceptual understanding.*  *T5*  *Advanced content is necessary, but not every hospital can implement it immediately. It depends on whether the hospital has the relevant cases, equipment, and team support. Without these supporting conditions, trainees are more likely to achieve conceptual updates rather than direct practical implementation.*  *M2*  *The training covered postoperative management of some complex skull base tumors and nursing care related to new technologies. However, our hospital does not have many such cases, so opportunities to use this knowledge in practice are limited. Nevertheless, this content did help me see the future direction of neurosurgical nursing.*  *S14*  *Whenever I learned something new during the training, I would think about whether it could be used in our department. In actual application, it was not always possible to transfer it exactly as it was taught. Instead, I adapted it according to the actual situation in our department and modified it into a process suitable for us.*  *S10*  *Although we do not have equipment for targeted temperature management, I drew on the training concepts, adjusted the frequency and approach of temperature monitoring, and optimized our current temperature management process.*  *S4*  *Our department mainly treats patients with brain metastases rather than primary gliomas. Therefore, I adapted the brain edema management and antiepileptic prevention procedures from the training and applied them to patients with metastatic tumors.*  *S5*  *Real transfer does not mean copying the training content exactly as it is. Rather, it means being able to adjust it according to the characteristics of patients and the resource conditions of one's own department.*  *T3*  *Some trainees adapted training cases into teaching cases for their own departments, or simplified complex processes into nursing guidelines suitable for their local settings. This is actually a very good form of transfer.*  *M3* |
| **Sub-theme: Training outcomes extended from individual care to specialty development** | *I adapted OSCE cases from the training, such as the management of postoperative intracranial pressure crisis, into departmental training materials. Now younger nurses are noticeably more confident when facing ICP alarms.*  *S7*  *After the training, I participated in revising the health education process for patients with brain tumors in our department, and I also incorporated some complication observation items into quality control checks.*  *S9*  *A particularly obvious change was that after returning to work, trainees were not only involved in clinical nursing, but also participated in departmental training, quality improvement, and specialty process development. This had a positive driving effect on improving the quality of specialty nursing in the department.*  *M4* |
| **THEME TWO: Influencing Mechanisms of Training Transfer Among Neurosurgical Specialist Nurses** | |
| **Sub-theme: Individual motivation and professional identity promoted training transfer** | *I participated in the training because I wanted to learn neurosurgical nursing more systematically, improve my own professional competence, and bring useful concepts and techniques back to my department to help solve practical problems.*  *S2*  *Previously, I paid more attention to postoperative vital signs and wound conditions. Now I pay more attention to patients' anxiety, functional recovery, and quality of life after discharge.*  *S13* |
| **Sub-theme: Contextualised and practice-oriented teaching promoted knowledge transformation** | *Our neurosurgical subspecialty system is relatively mature, and much of the training content was highly consistent with the needs of our department. Postoperative observation, complication identification, and rehabilitation guidance are used in our daily work, so they could be applied immediately after the training.*  *S8*  *Some complex neurosurgical diseases are not treated in our hospital. Even if I understood that part of the content, I basically had no opportunity to use it after returning to work.*  *S14*  *Brain tumor nursing should not only cover general neurosurgical content. It also needs to be linked to the nursing priorities for different tumor types and different surgical approaches; otherwise, it is difficult for trainees to connect what they have learned with specific patients after returning to work.*  *T7*  *A needs assessment should be conducted before training, and targeted teaching should be provided in a stratified manner. If a trainee's hospital only treats a dozen or so brain tumor patients a year, then teaching highly advanced treatment and nursing content in great detail is actually of limited significance.*  *T9*  *The management of neurosurgical emergencies cannot rely only on PowerPoint presentations. What is truly effective is allowing trainees to experience the entire simulated process, from identifying abnormal changes in a patient's condition to handling the situation, reporting it, and transferring the patient.*  *T6*  *If assessment consists only of theoretical and skills examinations, it cannot fully reflect whether trainees can apply what they have learned in real clinical scenarios. Case presentations, scenario simulation, OSCEs, nursing care plan design, post-training practice projects, and continuous evaluation are better able to reflect their comprehensive application of specialty care knowledge.*  *T10*  *If VR could be used to simulate awake craniotomy nursing or end-of-life communication scenarios, the teaching effect would be more realistic. However, the training base currently lacks the relevant conditions.*  *M1* |
| **Sub-theme: Organizational empowerment and teamwork provided implementation support** | *Some neurosurgical patients require support in rehabilitation, nutrition, and psychological care. If physicians and other team members cooperate, the specialist role of nurses can be demonstrated more easily.*  *S11*  *The care of neurosurgical patients involves surgeons, critical care, psychological intervention, nutrition, rehabilitation, and other areas. Whether the team is willing to collaborate directly affects whether specialist nurses can implement the training content in practice.*  *T2*  *When I carried out the project, leaders and colleagues were very supportive and made efforts to provide conditions for me to implement new skills and methods. The work progressed smoothly, and I was also highly motivated to translate the training content into practice.*  *S9* |
| **Sub-theme: Resource constraints and insufficient post-training support affected the sustainability of transfer** | *Some content requires equipment or information system support, but the current conditions in our department are limited, so we can only make some simple improvements first.*  *S12*  *The clinical workload is heavy. Most of the time, I can only complete routine treatment and nursing tasks first. I want to carry out specialist projects, but I do not have enough time or energy to further promote the new techniques and content learned during the training.*  *S15*  *Our hospital does not have an independent subspecialty ward. Patients are scattered across general neurosurgical wards, so it is difficult for me to implement the disease-specific management processes learned during training.*  *S10*  *The hospital does not have a palliative care team, so when patients with brain tumors enter the end-of-life stage, I cannot truly carry out the end-of-life communication learned during training.*  *S1*  *After returning from training, I had more responsibilities, including teaching, quality control, and research. However, there was no obvious change in performance appraisal or recognition, and over time this can affect motivation. If the hospital could provide clearer support in promotion pathways, performance incentives, or specialist nurse development, everyone would be more motivated.*  *S9*  *If the hospital cannot establish neurosurgical specialist nursing positions, differentiated performance incentives, and career development pathways, then the investment in training will eventually be lost, and nurses may also become burned out or even leave their posts.*  *T1*  *During the training, my mentor communicated with me every week and provided guidance. But after returning to my hospital, there was almost no one with whom I could discuss problems.*  *S3*  *After returning to work, communication was superficial, and we still lacked sufficient strength when conducting multicenter research. I hope there will be a platform for in-depth communication and continuous practice guidance.*  *S4*  *After the training ended, learning platforms and annual conferences were helpful to some extent, but systematic follow-up, feedback, and continuous guidance were still insufficient. In particular, support for continuously advancing post-training practice projects was lacking.*  *M4* |
| **Sub-theme: The unique neurosurgical context drove the expansion of nursing roles** | *After returning to work, I gradually became the default contact nurse for new patients with glioma. I coordinate MRI examination times, explain KPS scores to family members, and communicate between neurosurgery and radiotherapy departments.*  *S11* |
